# Clinical Utility of Diffusion Tensor Imaging and Fibre Tractography for Evaluating Diffuse Axonal Injury with Hemiparesis

**DOI:** 10.1155/2013/321496

**Published:** 2013-11-27

**Authors:** Ken Sugiyama, Takeo Kondo, Yoshimi Suzukamo, Yutaka Oouchida, Mari Sato, Hiroshi Watanabe, Shin-Ichi Izumi

**Affiliations:** ^1^Department of Physical Medicine and Rehabilitation, Tohoku University Graduate School of Medicine, 2-1 Seiryo-cho, Aoba-ku, Sendai 980-8575, Japan; ^2^Division of Physical Medicine and Rehabilitation, Tohoku Employees' Pension Welfare Hospital, 1-12-1 Fukumuro, Miyagino-ku, Sendai 983-8512, Japan

## Abstract

Although diffuse axonal injury (DAI) frequently manifests as cognitive and/or motor disorders, abnormal brain findings are generally undetected by conventional imaging techniques. Here we report the case of a patient with DAI and hemiparesis. Although conventional MRI revealed no abnormalities, diffusion tensor imaging (DTI) and fibre tractography (FT) revealed the lesion speculated to be responsible for hemiparesis. A 37-year-old woman fell down the stairs, sustaining a traumatic injury to the head. Subsequently, she presented with mild cognitive disorders and left hemiparesis. DTI fractional anisotropy revealed changes in the right cerebral peduncle, the right posterior limb of the internal capsule, and the right corona radiata when compared with the corresponding structures observed on the patient's left side and in healthy controls. On FT evaluation, the right corticospinal tract (CST) was poorly visualised as compared with the left CST as well as the CST in healthy controls. These findings were considered as evidence that the patient's left hemiparesis stemmed from DAI-induced axonal damage in the right CST. Thus, DTI and FT represent useful techniques for the evaluation of patients with DAI and motor disorders.

## 1. Introduction

Diffuse axonal injury (DAI) is widely accepted as one of the most common types of primary neuronal injury in patients with severe head trauma [1–4]. DAI results from damage to the white matter caused by unequal rotation and/or deceleration/acceleration forces acting on the brain parenchyma that stretch and injure axons [1, 2, 5, 6]. Patients with DAI present with various cognitive disorders in many cases and might present with motor disorders such as paralysis and ataxia. However, computed tomography (CT) and conventional magnetic resonance imaging (MRI) findings are often normal or reveal lesions which are poorly related to the disorders manifested [7–9].

Diffusion tensor imaging (DTI) has been recently developed as a useful tool for examining the organisation and structural integrity of the white matter [10, 11]. DTI uses MRI to quantify fractional anisotropy (FA), which characterises the degree and directionality of water diffusion (anisotropy) [10, 11]. FA values range from 1 to 0, with 0 being a completely symmetrical anisotropic medium, in which diffusion lacks any directionality (e.g., in water) and 1 representing maximum anisotropy, a medium in which diffusion is constrained to occur in one direction only [10, 11]. In addition, FA values have been reported to be useful in detecting various types of white matter damage, which might not be prominent on T1-weighted or T2-weighted MR imaging [8, 10, 11]. Furthermore, fibre tractography (FT) based on DTI data has been applied to visualise three-dimensional white matter fibres in the brain. FT utilises similarities between neighbouring voxels with regard to the shape and orientation of the diffusion ellipsoid. This technique can be used to visualise and study the connectivity and continuity of neural pathways in the white matter in the brain [11, 12].

There are some reports of the use of DTI for detecting DAI [8, 13–15]. Moreover, several reports have demonstrated an association between cognitive disorders and DAI lesions [16, 17], but few have demonstrated an association with paralysis. In this case study, we reported that it was possible to visualize the cause of paralysis evaluated by DTI and FT for a DAI patient with hemiparesis which shows no lesion in conventional MRI.

## 2. Case Report

**Figure 1:**
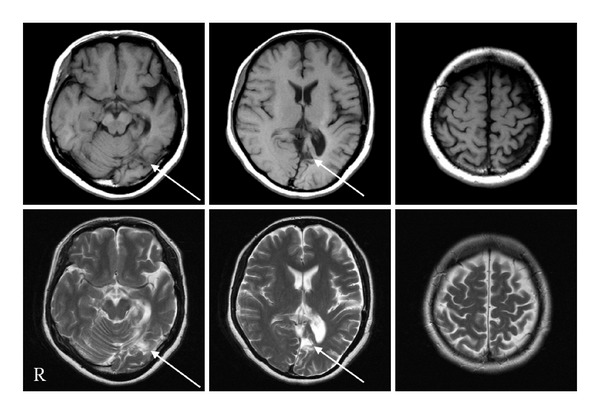
The upper sections are T1-weighted MR images, and the lower sections are T2-weighted MR images. A lesion was detected in the left occipital lobe (arrow), but no lesion speculated to be the cause of the left hemiparesis could be identified in the right cerebral hemisphere or the brainstem.

Our patient was a 37-year-old woman, who worked as an English teacher at a local high school. She fell down the stairs of the height of three meters and sustained a head injury. She exhibited a partial loss of consciousness scored 12 at (E3V4 M5) on Glasgow Coma Scale after the injury and was therefore transported to the emergency hospital. CT revealed a left acute subdural hematoma, which was treated using the craniotomy hematoma exclusion method. The patient's consciousness status improved after surgery, and she was transferred to our hospital for rehabilitation. On admission, the patient presented with a right homonymous hemianopsia, mild memory and attention disorders, and left hemiparesis. Although conventional MRI revealed a lesion in the left occipital lobe, no apparent cause of the left hemiparesis could be identified. The patient subsequently participated in a rehabilitation program which sufficiently improved her cognitive disorders to allow for a return to work 1 year later. Unfortunately, the left hemiparesis remained. The patient's left hemiparesis was scored as follows, according to the Brunnstrom staging: upper extremity IV, finger V, and lower extremity IV. Thus, she required a short leg brace and a cane for walking. Despite the stable cognitive status which allowed for a return to work, the patient never regained control of her left upper extremity and fingers and never regained a normal degree of locomotion. Two years after the injury, we performed additional MRI which revealed the same occipital lesion noted earlier, but no evidence could be determined regarding right cerebral hemisphere or brainstem abnormalities, which were speculated to be responsible for the left hemiparesis (Figure 1). Therefore, DTI and FT were used to identify the cause of the patient's left hemiparesis.

### 2.1. Diffusion Tensor Imaging

MRI was performed using a 1.5 Tesla imager (Signa Horizon LX CV/I, GE Medical Systems, Milwaukee, WI) and a conventional head coil to obtain DTI data. The DTI scan conditions were similar to those described previously [14, 17]. The DTI data obtained were transferred to an offline Windows PC with an Intel (R) Core (TM) i7 CPU processor (1.73 GHz) and 2046 MB memory. The data were then analysed using the diffusion TENSOR Visualizer (dTV) and VOLUME-ONE software (free software provided by the University of Tokyo Hospital, Tokyo, Japan; http://www.ut-radiology.umin.jp/people/masutani/dTV.htm).

At first, FA map images were obtained using dTV to measure the FA values. The FA values were measured using the region of interest (ROI) technique. Several sphere ROIs with a radius of 2.0 voxels were delineated on an FA map of the corticospinal tract (CST), which included the cerebral peduncle, the posterior limb of the internal capsule, and the corona radiata. After measuring the mean FA value of each ROI, we compared these to the corresponding values from five age-matched controls (average age, 35.2 years; range, 30–39 years). The Statistical Package for the Social Sciences (SPSS v. 11.0; SPSS Inc., Chicago, IL, USA) was used for statistical analysis, and a *P* value < 0.05 was considered statistically significant.

We conducted the CST evaluation by fibre tracking. The seed area was defined as a region of interest drawn around the cerebral peduncle, and the target area was defined as a region of interest drawn around the precentral gyrus. The stop criteria were set at FA < 0.18. The FT anisotropy information was colour-coded: red indicates high anisotropy, whereas orange indicates low anisotropy.

### 2.2. Result of the ROI-Based Analysis

Table 1 presents the results of the comparison outlined above. The mean FA values of the patient were significantly decreased as compared with those of healthy controls only on the right side of each ROI (*P* < 0.001); however, there were no significant differences on the left side. Further, bilateral comparison of the mean FA values from each ROI revealed the right-side values as significantly decreased in the cerebral peduncle (*P* = 0.001), the posterior limb of the internal capsule (*P* = 0.015), and the corona radiata (*P* < 0.001).

### 2.3. CST Fibre Tracking

**Figure 2:**
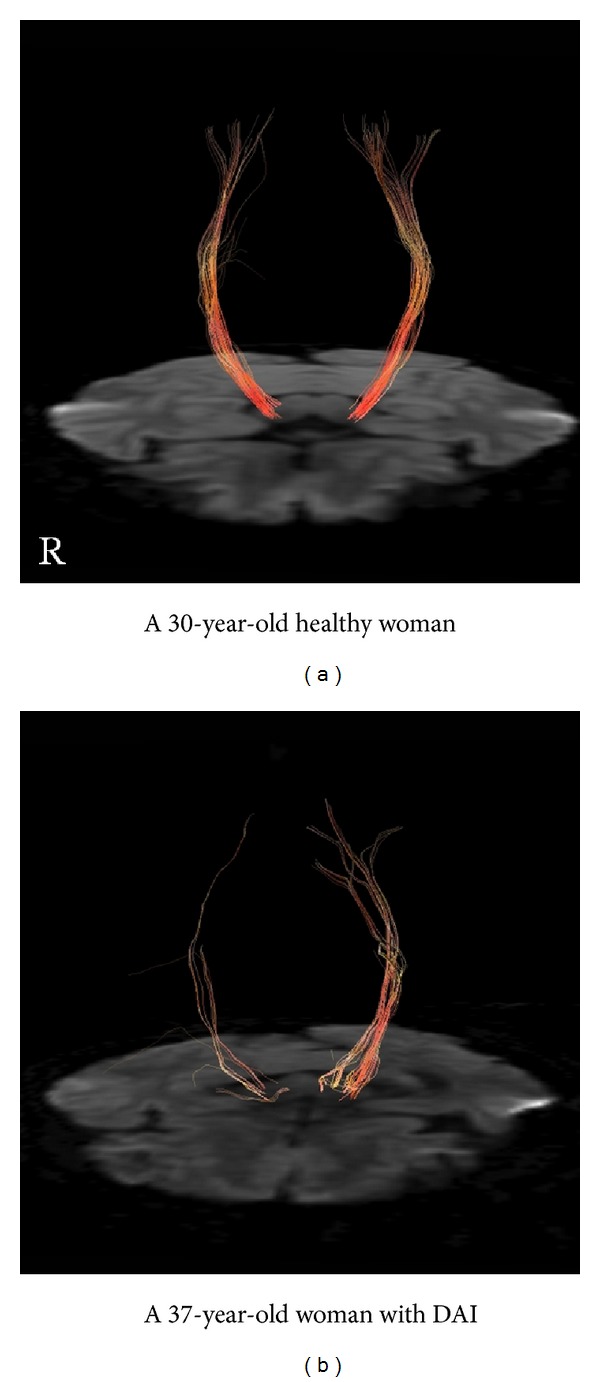
FT of CST from the seed area around the cerebral peduncle and the target area around the precentral gyrus. The right CST of the patient with DAI (tracked line, 118; drawn line, 6) is poorly depicted as compared with the left (tracked line, 252; drawn line, 34) and that of a healthy control (R: tracked line, 242; drawn line, 34. L: tracked line, 253; drawn line, 36).

On FT evaluation, the right CST of the patient with DAI was depicted less clearly (tracked line, 118; drawn line, 6) compared with that of the right CST of healthy controls (tracked line, 242; drawn line, 34; Figure 2). Furthermore, the depiction of the patient's right CST (tracked line, 118; drawn line, 6) was poor in comparison with that of the left CST (tracked line, 252; drawn line, 34; Figure 2).

## 3. Discussion

DAI is common among patients with traumatic brain injury (TBI) and often presents in association with cognitive and motor disorders. These disorders interfere with the ability to function independently and reenter society; therefore, DAI has become an issue of public concern. Despite such disorders, CT and conventional MRI typically fail to uncover any significant abnormalities in patients with DAI [7–9]. The objective evaluation of neural injury is necessary for the comprehensive evaluation of patients with DAI. Thus, there is an urgent need to establish the optimal imaging approach to achieve this end.

The unequal rotation and/or deceleration/acceleration forces which cause DAI also injure axons and induce localized bleeding [1, 2, 5, 6]. Over time, this damage to axons results in the development of gaps between fibres which were previously tightly packed. It is reported that this change facilitates water diffusion in the directions perpendicular to the axons, which in turn decreases FA observed in DAI [13, 18]. When comparing the right-side structures in our patient with those of age-matched controls, decreased FA values were observed in the cerebral peduncle, the posterior limb of the internal capsule, and the corona radiata. Moreover, bilateral comparison of the patient's brain structures revealed significantly decreased FA values in the cerebral peduncle, the posterior limb of the internal capsule, and the corona radiata. These findings are considered to quantitatively reflect the extent of axonal damage in each region. The FT images depicted the patient's right CST more clearly compared with those of her left CST as well as those of healthy controls. These findings are considered to represent the axonal fibres damaged by DAI. The decreased FA values revealed by the ROI evaluation as well as the poor CST depiction revealed by the FT evaluation were considered as evidence that DAI had injured the CST, thereby resulting in hemiparesis.

Some investigators have used DTI and FT to evaluate DAI [8, 13–17], but few reports have demonstrated relevance to motor disorders. Jang et al. [19] reported that they set ROI in the pons, the cerebral peduncle, the corona radiata, the medulla, and the posterior limb, which overlaps with the CST. Decreased FA values were observed in patients with DAI and motor weakness than in healthy controls. Ahn et al. [20] reported that CST depiction by FT was poorer in patients with DAI and right hemiparesis and/or quadriparesis than the in healthy controls. Here DTI and FT indicated that DAI-mediated CST damage could be the reason for our patient's hemiparesis. Nevertheless, the results provided by conventional MRI did not provide enough information for a valid conclusion. DTI and FT can be used to identify the causes of motor disorders in patients with DAI. These techniques allow the physician to visualise pathological changes, which in turn facilitates prognosis as well as the course to ultimate rehabilitation.

## 4. Conclusions

If conventional MRI fails to reveal any lesion which could be responsible for hemiparesis in a patient with TBI, evaluation using DTI and FT is useful. Further studies regarding DTI and FT and their correlation with the motor disorders in a larger number of patients with DAI will be necessary to confirm these findings.

## Figures and Tables

**Table 1:** Comparison between healthy controls and a patient with DAI and laterality of the mean FA values.

Region of interest	Healthy controls	Patient with DAI	*P* value*
CP			
R	0.51 ± 0.13	0.40 ± 0.13	<0.001
L	0.49 ± 0.14	0.49 ± 0.16	NS
*P* value**	NS	0.001	

PL			
R	0.59 ± 0.10	0.50 ± 0.13	<0.001
L	0.58 ± 0.10	0.56 ± 0.13	NS
*P* value**	NS	0.015	

CR			
R	0.60 ± 0.08	0.52 ± 0.09	<0.001
L	0.59 ± 0.06	0.60 ± 0.09	NS
*P* value**	NS	<0.001	

CP: cerebral peduncle; PL: posterior limb of the internal capsule; CR: corona radiata; R: right; L: left; NS: not significant.

*Comparison of healthy controls and patient with DAI by the independent *t*-test.

**Comparison of right and left by the paired *t*-test.
